# RE: Complete corporeal preservation clitoroplasty: new insights into feminizing genitoplasty

**DOI:** 10.1590/S1677-5538.IBJU.2021.0314

**Published:** 2021-05-05

**Authors:** Smail Acimi

**Affiliations:** 1 University of Oran Children's Hospital Canastel Department of visceral surgery Oran Algeria Department of visceral surgery, Children's Hospital Canastel, University of Oran, Oran, Algeria.

*To the editor,*

I have read with interest the manuscript written by Nicolas Fernandez et al. (1) on genital surgery in girls with Congenital Adrenal Hyperplasia (CAH) “Complete corporeal preservation clitoroplasty: new insights”. The clitoroplasty technique proposed by the authors is unclear: is it a disinsertion of the corpus cavernosum from the pubic bones, which are then separate from each other, then sutured to the pubic bones further than their initial insertion (a surgical technique similar to Kelly's procedure used in epispadias repair: complete detachment of insertions of the corpora cavernosa from the pubic bones, which is considered as dangerous and may expose in the repair of epispadias to catastrophic complications, such as partial or complete penile loss) or sliding of the two corpora cavernosa on the pubic bones after their separation as shown by the diagram D (Figure-3)?

In both cases, I would respectfully disagree with the authors: The two Figures 3A and 3C show neither of the two processes, but a simple separation of the two corpora cavernosa in the middle, creating two semicircles as shown by the black line created by the electric bistoury in the middle, and changing the angle of the image capture in figure 3C (lower than the other: Figure-3A) hides the insertion of the corpus cavernosum on the pubic bones. In addition, burying of the corpus cavernosum intact described by Lattimer in 1961, does not correct the malformation, but hides it. Moreover, this surgical technique is responsible for pain during erection in puberty and adulthood.

We perform a large number of feminizing genitoplasty per year and for more than 24 years. I think that the cosmetic results of the two patients shown in figure 4 do not correspond to the external genitalia of a girl and the results expected by the parents. One of the main reasons that lead the experts at the Chicago meeting in 2005 to recommend delaying surgical correction to adolescence is the high rate of poor cosmetic results in women treated in childhood for ambiguous genitalia. Reduction of the phallus should be performed as early as possible to allow the proper development of the patient's sexual identity. Feminizing genitoplasty should create the appearance of the external genitalia that corresponds to the gender. The esthetic result is considered very satisfactory when all four criteria are present (2, 3):

- Labia minora present with a free edge;
- Apparent part of glans <5mm;
- The area between two labia minora is covered by a red mucosa: this area should be covered by the wall of the urogenital sinus, never by perineal skin;
- Presence of two separate openings (vaginal and urethral).

The circumflex arteries of the phallus, lateral branches of the dorsal artery of the phallus, which supply the corpus spongiosum appear after the fourth year (4). The absence of these arteries during the first three years of life is an argument for performing clitoroplasty at an early age to avoid any risk of intraoperative and postoperative bleeding.

For more than 18 years, we use a variant of clitoroplasty (4, 5) characterized by excision of the distal and internal part of the corpora cavernosa after complete mobilization of the glans with its neurovascular bundle. This technique gives a significant and symmetrical reduction in the length and diameter of the corpus cavernosum. Reducing the diameter of the corpus cavernosum is an important step in the surgical correction of clitoromegaly because this part of the body is very thin in the woman. This can only be achieved after the complete release of the glans with its neurovascular bundle.

The Author
